# The effect of physical exercise on the anxiety of college students in the post-pandemic era: The mediating role of social support and proactive personality

**DOI:** 10.3389/fpsyg.2023.1128748

**Published:** 2023-03-15

**Authors:** Mengfan Liu, Bo Shi

**Affiliations:** ^1^School of Psychology, Northeast Normal University, Changchun, China; ^2^School of Physical Education, Northeast Normal University, Changchun, China

**Keywords:** post-pandemic era, physical exercise, anxiety of college students, social support, proactive personality

## Abstract

In order to study the current situation of the anxiety of college students in the post-pandemic era and the effect of physical exercise on anxiety, this study explores the influence of social support and proactive personality as mediating variables on the anxiety of college students from the perspective of physical exercise. Firstly, anxiety symptoms and anxious emotions are defined. Secondly, a questionnaire survey is conducted for a well-known university in a certain city, and different questionnaire scales are developed from the physical exercise, anxiety, social support, and proactive personality assessment of college students. Finally, the results of the survey are statistically analyzed to explore the relieving effect of physical exercise on anxiety. The results reveal that there is a significant gender difference in the level of physical exercise, and the amount of physical exercise of the male students is generally higher than that of female students. In addition, the intensity, time, and frequency of exercise of male students are more than that of female students, but there is no obvious difference between them and whether they were only children. Physical exercise habits, social support, proactive personality traits, and anxiety of college students have a significant correlation. Through the analysis of the chain mediation effect, Ind2 (0.0140) is the largest coefficient among the three paths, indicating that the path of influencing social support through physical exercise habits, followed by affecting proactive personality traits, and then impacting anxiety has the strongest explanatory force. According to the results, strategies to relieve the anxiety of college students are given. This study can provide a reference for the research on the methods to alleviate their anxiety under the influence of the epidemic.

## 1. Introduction

Post-pandemic era is a state of people’s production and life after the outbreak of the Corona Virus Disease 2019 (COVID-19) (Petersen et al., 2019; Tang et al., 2022; Zheng et al., 2022). In the early days of a post-pandemic era, when the number of infections increases rapidly, there may be some confusion and panic in the short term. However, as people gradually adapt to and accept the situation, they will reach a new steady state and find a new balance between epidemic prevention and economic development (Siying et al., 2021; Mo and Ming, 2022; Yang et al., 2022). Because of the epidemic, many people’s lives have changed, and it has triggered a lot of new thinking, such as the advantages and disadvantages of online and offline, the severe damage caused to various industries during the epidemic, the length of the recovery period, and so on, will change many people’s lives (Huang et al., 2021). College students are no exception, with delayed start times and online teaching becoming routine. Many foreign students cannot study abroad normally due to the epidemic (Rogowska et al., 2020). Learning efficiency has plummeted, and there is no way to go to the library, laboratory, and off-campus internship. Normal campus life has been greatly changed. For college students who are studying or about to enter social work, the initial feeling of freshness has gradually changed to a psychological state of anxiety and depression (Bamber and Morpeth, 2019; Wang et al., 2020; Watt et al., 2020). However, since the environment has been such, the best way to cope is to seek self-regulation and rely on the support of the surrounding society to achieve their academic goals.

With epidemic prevention and control normalized, college students’ extracurricular activities have been significantly reduced, and all off-campus activities have been canceled. Nucleic acid testing and vaccination have also become “frequenter” in the daily life of college students, which makes people understand the importance of physical health. Anxiety is produced by the accumulation of adrenaline, but a certain intensity of aerobic exercise can consume the adrenaline of the human body, thus achieving the purpose of relieving anxiety (Huang et al., 2018; Dominski and Brandt, 2020; Lee et al., 2021). Regular exercise can not only help people keep fit and increase vitality, but also improve their appearance and build up self-confidence (Zeng et al., 2018; Saeed et al., 2019; Grasdalsmoen et al., 2020). What is more, during the process of exercising, exercise also helps to release the tension of anxiety, so that both the body and mind are greatly relaxed. As such, moderate physical exercise has a positive effect on relieving the anxiety of college students (Goel et al., 2018; Ren and Li, 2020; Watt et al., 2022).

Moreover, after college students have anxiety, depression, and other unhealthy psychological states, often accompanied by a large number of negative emotions accumulation, in addition to physical exercise, their self-adjustment ability and social support from the surrounding environment also have a positive effect on easing anxiety (Rith-Najarian et al., 2019; Chang et al., 2021; Ren et al., 2021). That is, the mediating role of external the environment in relieving anxiety is considered. Thereupon, it is of practical significance to study the influence of physical exercise on the anxiety of college students in the post-pandemic era.

Although there have been many similar research conclusions, there are still some limitations. For example, they do not take into account the specific mediating effect of college students’ social support and personality traits on the research topic under specific circumstances, such as the COVID-19 pandemic. Thus, the questionnaire survey and comparative study are adopted. Firstly, this study reveals the impact of the post-epidemic era on various industries and the living situation of college students. It is hypothesized that there is a correlation among college students’ physical exercise habits, anxiety, and mediating variables (social support and proactive personality). Secondly, different questionnaire scales are developed according for college students to explore the easing effect of physical exercise on anxiety. Besides, the results of the survey are analyzed statistically. Finally, according to the results, strategies and suggestions to relieve the anxiety of college students are put forward. This study can alleviate the anxiety of college students and help them maintain healthy psychological conditions.

## 2. Literature review

This epidemic can be said to have had the largest and most serious impact in all mainstream countries since the beginning of the new century. In the foreseeable post-pandemic era, the post-western world is bound to undergo a larger-scale ideological transformation. “Post-pandemic era” can also be understood as the era after the epidemic, which affects people’s consumption habits, economy, culture, education, and others (Lin, 2022; Yan et al., 2022; Zhang, 2022). This has a great impact on college students, and even many students even suffer from an anxiety disorder, which is a serious mental illness. As anxiety causes changes in body function, it is generally accompanied by transformations in physiological and sports indicators. Many scholars give their own answers to this research.

For example, Johnston et al. (2021) conducted a 12-week quasi-experimental study aimed at evaluating the effectiveness of team sports in relieving depression, anxiety, perceived pressure, and poor sleep quality among college students. Data were collected by questionnaires before and after the test. The results suggested that team sports may help reduce depression and poor sleep quality among college students. However, physical exercise alone may not help improve anxiety and perceived stress. Siyal et al. (2021) studied how inclusive leaders can cultivate employees’ innovative behaviors and creativity by drawing on the social communication theory. Glavin et al. (2022) investigated the relationship between exercise, sleep, and mood of male and female college students. Students (*N* = 866, 19.6 ± 1.4 years old, 38.7% female) were recruited from campus recreation facilities to complete demographic, Pittsburgh Sleep Quality Index (PSQI), mood (Patient-Reported Result Measurement Information System), and exercise questionnaires. The results found that women went to bed earlier, slept less efficiently, and had higher levels of anxiety and depression than men (*p* < 0.05). Lv and Kumar (2020) pointed out that connecting infrastructure closely related to people through the network plays an important role in the wide use of the network. Deng et al. (2020) conducted a web-based survey through snowball sampling to collect demographic data, mental health status, sports-related lifestyle, and problems related to online sports of university students in Wuhan City. The results denoted that mental state was remarkably correlated with regular exercise and sufficient exercise time. Ghrouz et al. (2019) explored a cross-sectional among Indian college students in which all participants completed three questionnaires: the Hospital Anxiety and Depression Inventory, the International Physical Activity Questionnaire-Brief Form, and the PSQI. The study found that there was a prominent correlation between physical activity level, sleep quality, and mental health.

In addition, the amount of exercise also has a great impact on the physiological level. For example, St Clair Gibson et al. (2018) pointed out that both central (brain) and peripheral (physiological system of the body) control mechanisms, or a combination of these mechanisms, have been supported in the field of exercise science. Therefore, both psychological and physiological driving forces are based on the steady-state principle, and their relative activities are regulated by dynamic negative feedback activities as the basic general operating controller. Silva et al. (2021) studied the physiological and perceptual responses to aerobic exercise with and without blood flow restriction and high-intensity intermittent exercise, demonstrating the potential benefits of aerobic exercise and blood flow restriction on physical and mental health. Azizi et al. (2021) examined that training under normoxia and hypoxia conditions can be an effective factor in improving complications in overweight men through its beneficial effects on iridin levels and insulin resistance. The results demonstrated that exercise was very helpful to improve the physiological state of the human body. Jones et al. (2018) proposed that academic pressure is the biggest variable of college students’ anxiety, followed by economic pressure, family support, and peer support. The influence of social demographic variables is very small, indicating that different types of students generally have anxiety, which has a large impact on the normal life of students and must be taken seriously.

In summary, existing studies have shown that different kinds and degrees of physical exercise have significant effects on the relief of poor sleep quality, anxiety, depression, and other emotions. However, the influence of the surrounding environment on the psychological state of college students is not taken into account. Most studies on the psychological state of college students under normal circumstances are less in the context of the epidemic. Consequently, the relationship between physical exercise and the anxiety of college students under a specific research background is studied, which can provide a new research idea for alleviating their anxiety.

## 3. Investigation of the current situation of the anxiety and physical exercise of college students in the post-pandemic era

### 3.1. The definition of anxiety

The word anxiety initially means that when an individual is faced with something indecisive, he or she cannot make a decision quickly, showing the inner feeling (Knowles and Olatunji, 2020). Anxiety is an emotional problem, including worry, tension, fear, and many other uncomfortable emotional feelings. It is an instinctive emotion, and everyone will have anxiety. Anxiety occurs when people are in a stressful mental state or are stimulated (Patterson et al., 2021). In fact, not all anxiety is bad. Proper stress and anxiety can motivate people to think and solve problems positively.

The scientific name of anxiety disorder is anxiety neurosis (John Lothes et al., 2021). Its main symptom is anxiety, which is a neurosis with anxiety as the main clinical manifestation. It is often accompanied by motor discomfort and physical discomforts, such as insomnia, increased heart rate, excessive sweating, loss of appetite, frequent urination, and urgent symptoms (Sprung and Rogers, 2021). Anxiety disorder is a general term that can be subdivided into the following diverse types of anxiety: general anxiety, social panic disorder, simplex phobia, panic disorder, agoraphobia, obsessive–compulsive disorder, hypochondria, etc. Different anxiety disorders show various behaviors, so the treatment is not the same, but basically will have an impact on our lives, and even affect people’s normal social functions, such as being unable to study, work, do housework or even the simplest of tasks cannot be done on their own.

Severe anxiety symptoms often involve motor restlessness, such as restlessness. There will also be rubbing hands and feet, both upper and lower limbs shaking (tremor), and then serious symptoms such as wandering, and pacing back and forth. Anxiety symptoms are also often accompanied by sleep problems, mainly manifested in difficulty falling asleep. Besides, people also show inattention, focus only on their own discomfort symptoms, do not care about others, and may even be overly demanding of others to care for them. Anxiety is a normal state of mind for almost everyone when faced with difficulties or dangers. It can be said that anxiety is a positive stress instinct, and appropriate anxiety can help people summon up the courage to solve an impending crisis. However, anxiety symptoms occur when the level and duration of anxiety exceed a certain range, which can have the opposite effect, such as preventing people from dealing with the crisis before them and even interfering with normal life.

### 3.2. Questionnaire survey respondents and method

This study takes freshmen to seniors of a well-known university in a city as the research respondents to investigate their physical exercise, mental health (such as anxiety), social support, and personality assessment of college students in the post-pandemic era. Additionally, the mediating effects of different social support and proactive personality of college students on anxiety are analyzed. Consent is obtained from the school, teachers, and students themselves before the investigation, and students are asked to sign informed consent and privacy protection agreement to ensure that the investigation is conducted without controversy.

Based on previous studies, the following hypothesis is proposed:

*Hypothesis 1:* There is a correlation among college students’ physical exercise habits, anxiety, and mediating variables (social support and proactive personality).

**Figure 1:**
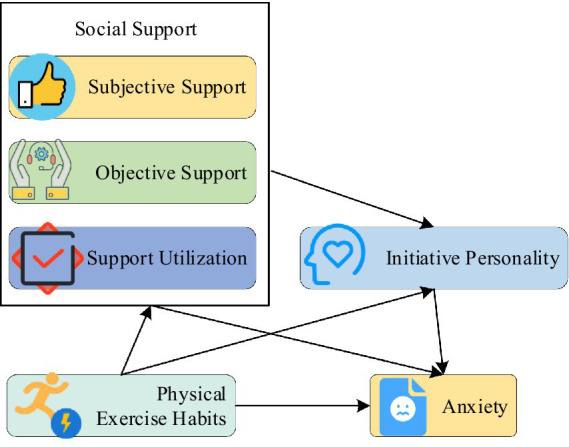
Chain mediating hypothesis model.

*Hypothesis 2:* The social support and proactive personality of college students have a chain mediating effect on the influence of physical exercise habits on anxiety. The hypothesis model is displayed in Figure 1.

The questionnaire is divided into four parts. Part 1 is the survey of college students’ physical exercise habits under the influence of the epidemic. The Physical Activity Rating Scale-3 (PARS-3) was adopted, which was compiled by Japanese scholar Masao Hashimoto in 1990 and revised by Chinese scholar Deqing Liang in 1994. The PARS-3 measures the physical activity level of individuals from three dimensions: the frequency, intensity, and time of exercise. Among them, the frequency and intensity of exercise are scored by 1–5 points from levels 1–5, and the time of exercise is scored by 0–4 points from levels 1–4. The physical activity level of the respondents is calculated according to the equation of the amount of physical activity = exercise intensity × (exercise time − 1) × exercise frequency. The highest score for exercise is 100, and the lowest score is 0. Evaluation range of physical exercise amount: a small amount of exercise ≤19 points; Moderate exercise: 20–42 points; Large exercise ≥43 points. The higher the score, the stronger the level of physical activity, and the greater the amount of physical activity of the individual. After the reliability analysis of the scale in this study, Cronbach’s alpha coefficient is 0.758 > 0.7, indicating that the scale is highly reliable and suitable for investigation.

Part 2 explores the anxiety of college students, which is carried out by Generalized Anxiety Disorder (GAD-7) (Spitzer et al., 2006). The GAD-7 was compiled by Spitzer et al., consists of 7 items, and uses the 4-point scoring method, with 0 to 3 points, respectively. The total score of the GAD-7 ranges from 0 to 21. A score of 0 to 4 refers to no symptoms of anxiety, 5 to 9, 10 to 14, and 15 to 21 indicate mild, moderate, and severe anxiety symptoms.

Part 3 studies the social support situation of the students in the university, which is performed by the Social Support Rating Scale (SSRS). The scale was compiled by Xiao Shuiyuan et al., consisting of 10 items in 3 dimensions (subjective support, objective support, and support utilization). 1–5 and 8–10 items were scored with 4 points and assigned 1–4 points, respectively. 6–7 items are scored with 2 points, and they are assigned 0–1 points, respectively. The total score of the scale ranges from 12 to 66, with scores from 0 to 22, 23 to 44, and 45 to 66 indicating low, moderate, and high levels of social support, respectively.

Part 4 investigates the personality characteristics of college students. This part adopts the proactive personality scale developed by Seibert et al. (1999). This scale was mainly revised by referring to the scale proposed by Thomas et al. (1993). The original 17 items were reduced to 10 items. The reliability coefficient of this scale is 0.89, which has good measurement reliability and adopts Likert’s five-point scoring method. One refers to completely inconsistent, and so on, and 5 stands for very consistent. The higher the score, the higher the level of proactive personality.

After the questionnaire design was completed, a formal questionnaire survey was conducted. A total of 600 questionnaires were distributed in the school. According to the filling situation of the questionnaire, such as whether to complete all the items and whether to fill in in a distinguishable way, 23 questionnaires that did not meet the requirements were screened out. Five hundred seventy-seven questionnaires were collected on-site, with a recovery rate of 96.17%. Then, SPSS24.0 was used for data test analysis, including independent sample T-test of gender and whether the only child, correlation analysis of physical exercise and anxiety, regression analysis, etc. Next, statistical analysis of the result data and the mediating effects of social support and proactive personality were carried out.

To further study the internal relationship between college students’ physical exercise habits, anxiety, and chain mediating variables (social support and proactive personality traits), the mediating test procedure is adopted here. At present, the product of coefficients is tested directly by the Bootstrap method, which is a method of repeated sampling from samples. In this study, the model of Process v3.4 plug-in in SPSS (Figure 1) will be employed to conduct a regression test of chain mediating variables in physical exercise habits and anxiety.

## 4. Results and discussion

### 4.1. Descriptive statistical results of the questionnaire survey

**Figure 2:**
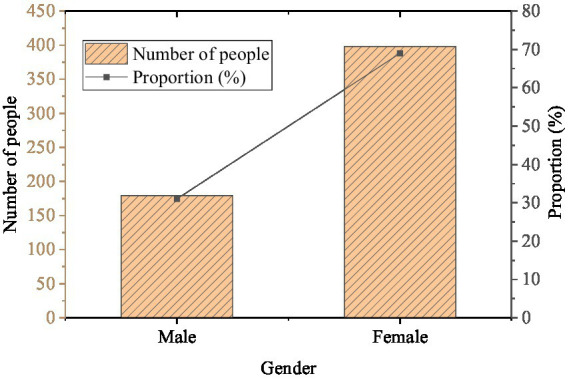
Gender distribution (*N* = 577).

**Figure 3:**
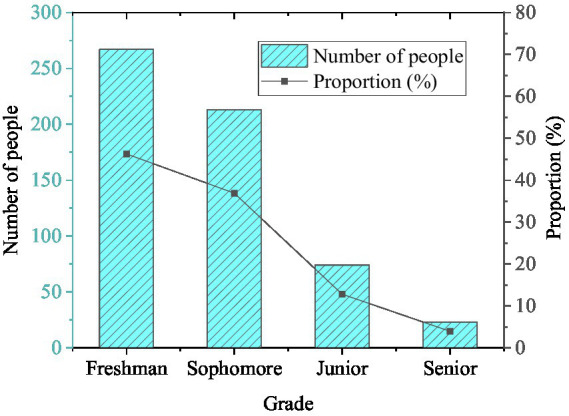
Grade distribution (*N* = 577).

**Figure 4:**
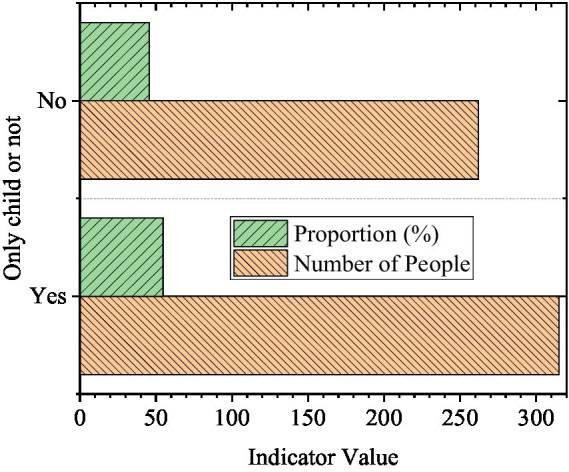
Situation of only child (*N* = 577).

The statistical results of gender, grade, and whether the only child of the students participating in the questionnaire is exhibited in Figures 2–4.

In Figure 2, there are many female students among the respondents, accounting for 68.98%, and the proportion of male students is 31.02%. Figure 3 shows that there are 267 freshmen, 213 sophomores, 74 juniors, and 23 senior students in the survey, accounting for 46.27, 36.92, 12.82, and 3.99%, respectively. Figure 4 demonstrates that the number of only children and non-only children is 315 and 262, accounting for 54.59 and 45.41%. It can be found that there are more female respondents, and most of them are freshmen and sophomores. The number of only children is slightly more than that of non-only children. This is related to the ratio of males to females in this school. Besides, the juniors and seniors are under great pressure from schoolwork and have more internships and less free time, so the questionnaire ratio is affected.

**Figure 5:**
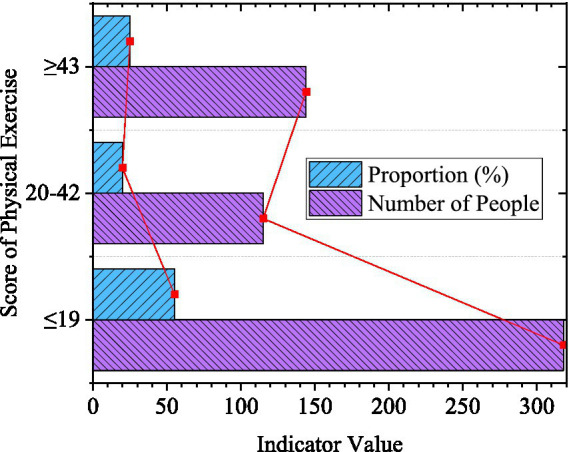
Physical exercise of college students (*N* = 577).

For the score of physical exercise, a score below 19 as mentioned above means a small amount of exercise; A score between 20 and 42 indicates moderate physical activity; A score greater than 43 indicates heavy exercise. The higher the score, the stronger the level of physical activity, and the greater the amount of physical activity of the individual. The statistical results are demonstrated in Figure 5.

In Figure 5, the number of college students who score 19 points or below is 318, accounting for 55.11%, more than half of the total number. The number of students who score 20–42, and 43 or above points is 115 and 144, accounting for 19.93 and 24.96%. It can be found that more than half of college students in the post-pandemic era have a small amount of exercise, and less than one-fifth of those who maintain high exercise. On the one hand, it is restricted by the activity site during the epidemic; on the other hand, the epidemic prevention and control requirements do not allow a large number of people to gather, so the places and times of college students’ activities are also limited.

**Figure 6:**
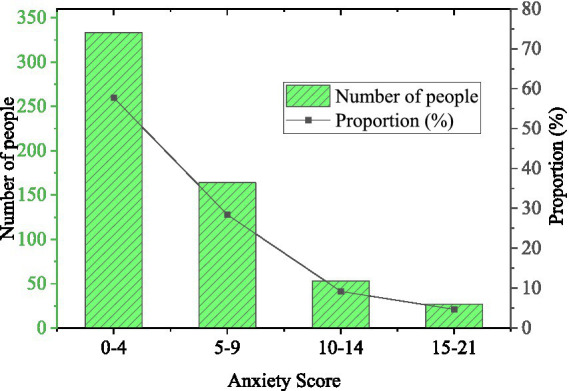
Anxiety scores of college students (*N* = 577).

Regarding the scores of anxiety of college students during the epidemic period, the scores ranged from 0 to 4, 5 to 9, 10 to 14, and 15 to 21 standing for no symptoms, mild, moderate, and severe anxiety symptoms. The details are plotted in Figure 6.

Figure 6 signifies that the anxiety score of college students ranges from 0 to 4, that is, 57.71% have no anxiety symptoms. The ratio of students with mild, moderate, and severe anxiety symptoms on a score of 5–9, 10–14, and 15–21 is 28.42, 9.19, and 4.68%. It can be seen that college students have different levels of anxiety in the era of the epidemic, and the proportion is nearly half. Thus, it needs to be highly valued, which will inevitably affect the physical and mental health, life, and study of college students.

**Figure 7:**
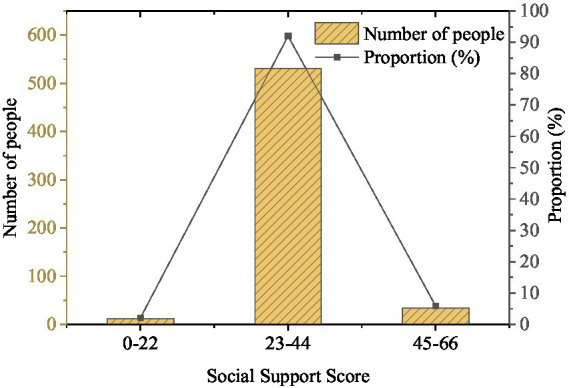
Social support scores of college students (*N* = 577).

The social support received by college students is presented in Figure 7.

Figure 7 expresses that the total score of support covers three dimensions: subjective support, objective support, and support utilization. The scores ranging from 0 to 22, 23 to 44, and 45 to 66 indicate that the level of social support is low, medium, and high, accounting for 2.08, 92.03, and 5.89%, respectively. It means that the level of social support received by most college students is at the medium level.

### 4.2. Data analysis

SPSS24.0 is employed to test the difference in college students’ physical exercise levels and the results are outlined in Table 1.

**Table 1:** Division of physical exercise of college students.

Classification	*M*	df	*F*	Sig
A small amount of physical exercise	8.03	2	1489.26	0.000
Moderate exercise	29.03			
A large amount of exercise	68.90			
Total	27.41			

*M* represents the average value, the same as below.

Table 1 denotes that the average value of the low-exercise group is 8.03, which is significantly lower than the overall average value of 27.41, with a difference of about 20 points. The average value of a large amount of exercise group is nearly 40 points higher than the general level, the degree of freedom is 2, the *F* value is 1489.26, and sig < 0.01, illustrating that there are significant differences between diverse levels of exercise to classify physical exercise habits. Beyond that, the results of exercise with different exercise habits are also markedly different.

The independent sample *T*-test results of different physical exercise levels in gender and whether they are only children are signified in Table 2.

**Table 2:** Test results of differences in physical exercise of college students on gender and whether they are only children (*N* = 577).

	$\bar{X}$	*SD*	*T*	Sig
Male student	3.4258	1.069	3.265	0.000
Female students	3.1259	1.245		
Only children	3.3251	1.169	−1.425	0.859
Non-only children	3.3521	1.172		

*SD* stands for standard deviation, the same as below.

In Table 2, there is an obvious difference in the level of physical exercise between the gender, Sig < 0.01, and the amount of physical exercise of the male is generally higher than that of females, exercise intensity, time, and frequency of males are more than that of females. However, there is no significant difference between them and whether they are only children (Sig > 0.01). This is because what men usually play in society is a kind of physical beauty with a strong physique, so male students will choose to change their external image and enhance their personal charm by participating in sports, and college students are no exception. It illustrates that the physical exercise habits of college students are related to gender, and there are differences between boys and girls. However, there is no obvious relationship with whether it is an only child, indicating that the family organizational structure does not have much influence on the physical exercise situation itself.

Table 3 presents the difference in the anxiety degree of college students under different levels of physical exercise habits.

**Table 3:** Test results of anxiety level difference under different conditions of physical exercise.

		Difference of average value	Standard error	Sig
A small amount of physical exercise	Moderate exercise	−0.410	0.974	0.000
	A large amount of exercise	10.891*	1.096	0.000
Moderate exercise	A large amount of exercise	11.263*	0.974	0.000

* indicates significant difference.

In Table 3, there is a prominent difference in the anxiety level between the group with a small and a large amount of physical exercise (Sig < 0.01); there is a remarkable difference in the anxiety level between moderate and large exercise groups, while there is no significant difference between small and moderate exercise groups. This indicates that only a high amount of physical exercise habit can significantly affect the level of anxiety. Because anxiety is not a short-term state of anxiety, but a long-term existence in the personality structure of individuals, so a short period of physical exercise cannot have a significant impact on the improvement of anxiety.

The correlation test among the four variables of physical exercise habit, anxiety, social support, and proactive personality is portrayed in Table 4.

**Table 4:** Correlation test results of physical exercise habits, anxiety, social support, and proactive personality.

	Physical exercise habits	Social support	Proactive personality	Anxiety
Physical exercise habits	1			
Social support	0.225**	1		
Proactive personality	0.625**	0.596**	1	
Anxiety	−0.539**	−0.269**	−0.569**	1

** refers to an obvious difference at the 0.01 level.

In Table 4, there is an obvious correlation between college students’ social support and physical exercise habits, and the Pearson coefficient is 0.225. It refers that these two are positively correlated, the higher the level of social support, the better the physical exercise habit; the worse the level of social support, the worse the physical activity habits. There is an evident correlation between physical exercise habits and proactive personality, and the Pearson coefficient is 0.625. It means that they are positively correlated, the higher the degree of the proactive personality trait, the better the physical exercise habit; The worse the degree of the former, the worse the latter. There is a distinct correlation between physical exercise habits and anxiety of college students, and the Pearson coefficient is −0.539. The results illustrate that physical exercise is negatively correlated with the anxiety of college students. The better the physical exercise habit, the lower the anxiety level of college students. The worse the former, the higher the latter. This is consistent with the previously proposed hypothesis 1.

Physical exercise is taken as the independent variable and anxiety is the dependent variable for regression analysis to predict the relationship between the two. The results are revealed in Table 5.

**Table 5:** ANOVA test of physical exercise habits on anxiety.

	Sum of squares	Degree of freedom	Mean square	*F*	Sig	*R* ^2^
Regression	29659.36	1	29659.36	58.96	0.000	0.099
Residual	295686.55	559	519.36			

ANOVA stands for analysis of variance. a. Dependent variable: physical exercise habits; b. Predictive variable: (constant), anxiety.

Table 5 shows that sig < 0.01 and *F*-value is 58.96, indicating that the above model passes the significance test and the linear regression model is established. Physical activity can explain 9.9% of anxiety. Then the independent variable X: physical exercise habits; the dependent variable Y: anxiety; mediating variable M1: social support; the mediation variable M2: proactive personality traits are applied to the Bootstrap method of process plug-in to test whether the model is valid. The results are expressed in Table 6.

**Table 6:** Regression analysis of physical exercise habits, anxiety, social support, and proactive personality.

Dependent variable	Independent variable	Partial regression coefficient			*R*	*R* ^2^	*p*
		Coeff	*SE*	*t*			
Social support		32.5692	2.3659	15.9215	0.6635	0.4536	0.0000
	Physical exercise habits	0.5369	0.0254	21.6351			
Proactive personality		63.3695	1.5692	41.2596	0.5526	0.3098	0.0000
	Social support	0.3023	0.0025	11.2631			
	Physical exercise habits	0.3695	0.0269	0.1598			
Anxiety		12.6584	0.5236	25.6321	0.6459	0.4563	0.0000
	Social support	−0.0245	0.0059	−4.5961			
	Proactive personality	−0.0365	0.0069	−16.3256			
	Physical exercise habits	−0.5399	0.0036	−0.9962			

Table 6 implies that in the regression analysis of the effect of physical exercise habits on social support, *R*-value is 0.6635, the *R*^2^ value is 0.4536, *p* < 0.01, with statistical significance. The *R*-value of the regression analysis of physical exercise habits on proactive personality is 0.5526, and the *R*^2^ value is 0.3098, *p* < 0.01, which is statistically significant. Social support and proactive personality traits have a significant negative effect on anxiety, the higher the social support, the lower the anxiety; the higher the degree of proactive personality, the lower the level of anxiety. These results suggest that proactive personality traits and social support have mediating effects on anxiety. The mediation pathway test is then performed, as illustrated in Table 7.

**Table 7:** The mediation pathway test.

Indirect effect(s) of *X* on *Y*				
Effect	Boot	SE	BootLLCI	BootULCI
Total:	0.0265	0.0036	0.0218	0.0335
Ind1:	0.0125	0.0028	0.0059	0.0154
Ind2:	0.0140	0.0018	0.0123	0.0159
Ind3:	0.0001	0.0024	0.0049	0.0059

In Table 7, Ind1 means X → M1 → Y, Ind2 refers to X → M1 → M2 → Y, Ind3 stands for X → M2 → Y. The interval of the overall mediation effect is not 0, showing that the overall mediation effect is valid. There are three paths in the model. Ind1: physical exercise habits → social support → anxiety; Ind2: physical exercise → social support → proactive personality traits → anxiety; Ind3: physical exercise habits → proactive personality traits → anxiety. The coefficient of Ind2 is 0.0140, which is the largest among the three paths, illustrating that the path of impacting social support through physical exercise habits, then affecting proactive personality traits, and then influencing anxiety is the strongest explanation. Furthermore, proactive personality and social support can also act as separate mediating variables to affect levels of anxiety, and when both levels are high, the mediating effect on anxiety level is the most obvious, which verifies hypothesis 2.

### 4.3. Discussion

On account of the above research results, all the hypotheses established above have been verified. The different situations of college students’ physical exercise will indeed have some help to the environment of anxiety. This is similar to the findings of Frederiksen et al. (2021), who suggested that physical exercise was promoted as a means to enhance the impact of cognitive behavior therapy (CBT) on anxiety disorders. Physical exercise seems to reduce anxiety through mechanisms other than CBT, some of which may also enhance the effects of psychotherapy. Evidence from a systematic review of randomized controlled trials by Kandola et al. (2018) suggested that exercise training can reduce symptoms of anxiety and stress-related disorders such as post-traumatic stress disorder, agoraphobia, and panic disorder. Herzog et al. (2022) examined the effect of a single acute exercise session on state anxiety in subclinical samples of moderate to high anxiety sensitivity, exploring potential mediating factors including self-efficacy, self-esteem, mindfulness, subjective vitality, rumination, and emotional competence. They found that one acute exercise may not be sufficient to reduce state anxiety in subclinical individuals with moderate to high anxiety sensitivity. However, positive influence and subjective vitality may be two mechanisms that explain the beneficial effects of exercise on anxiety.

Furthermore, Naghavi et al. (2018) aimed to investigate the effects of cognitive and physical training on anxiety in older adults. Sut Txi et al. (2022) investigated the prevalence of emotional distress in athletes during Covid-19 in Malaysia. There were no significant differences between levels of stress, anxiety, and depression based on age, gender, and exercise category (individual *VS* team sports). More female athletes reported severe depression (4.19%) than male athletes (3.13%). This finding recommends further assessment, monitoring, and treatment plans for athletes, especially female athletes, to ensure their mental health and emotional well-being during the Covid-19 pandemic. It can be seen that this study has certain reliability and research basis.

## 5. Strategies for relieving anxiety of college students in the post-pandemic era

Based on the above results, it is necessary to establish a subjective awareness of physical exercise for the groups troubled by anxiety and depression, and regular exercise is very important. Moreover, physical exercise can enable individuals to effectively manage their own pressure and ability to bear it and has a relatively obvious effect on relieving individual anxiety, depression, and other negative emotions. Hence, for college students who have anxiety, irregular physical exercise is a better way to relieve it. Specific measures are as follows:

1) Moderate-intensity physical exercise

As can be seen from the above research results, only a high amount of physical exercise habit can significantly affect the level of anxiety. Thereupon, high-intensity physical exercise (overall score of physical exercise is above 43 points), such as aerobics, can improve anxiety and reduce the impact of bad emotions on life and study. For students with high scores of anxiety, moderate-intensity aerobic exercise can be adopted to reduce anxiety; for students who want to reduce anxiety for a short time, high-intensity exercise can be used to quickly relieve anxiety.

2) Seek help from the supporting forces of the surrounding society

External support from family, school, and social environment is of great help to relieve the anxiety of college students. Therefore, these external forces need to carry out regular screening of students’ anxiety symptoms, so as to timely discover these high-trait anxiety groups and conduct psychological intervention and exercise interventions for them. In particular, within the scope of epidemic prevention and control, schools need to organize regular and quantitative sports activities in a small range, and open sports venues, such as school football fields, sports equipment, and other sports projects to prevent anxiety.

3) Strengthen psychological quality, face anxiety directly, and strengthen body and heart at the same time

In the final analysis, anxiety is an individual’s own psychological emotions in the face of external emergencies or a new environment. Therefore, if college students want to maintain good mental health, what they need to do most is to strengthen their own psychological quality, such as developing their own interests, reading books, keeping themselves busy, making study and life plans, and maintaining an optimistic and positive attitude toward the uncontrollable situation (the change of lifestyle brought by the epidemic).

## 6. Conclusion

Since the outbreak of COVID-19, the life, study, and work of college students have been affected to varying degrees, and it is inevitable that they will have anxious emotions and even form anxiety disorders. Thereby, the strategies for easing the anxiety of college students in the post-pandemic era are studied. Firstly, anxious emotion and anxiety disorder are defined. Due to diverse psychological anxiety levels, they are treated differently. Secondly, a questionnaire survey is carried out for a well-known university in a certain city. Various questionnaire scales are developed based on the physical exercise, anxiety, social support, and proactive personality evaluation of college students. Finally, according to the results of the survey, it is found that a certain degree of physical exercise has a certain effect on relieving the anxiety of college students, and it is closely related to the social support and personality traits of college students themselves. The conclusion of this study can provide practical significance for the exploration of the mental health of college students. The limitation of this study lies in the limited number of samples collected due to the limitations of conditions, so it cannot fully analyze the psychological conditions of all college students. In addition, this survey mainly focuses on the anxiety of college students, without paying too much attention to other mental health problems, such as depression and tension. Thereupon, it is planned to expand the research scope, further increase the number of samples collected, and expect to obtain more in-depth research results.

## Data availability statement

The original contributions presented in the study are included in the article/supplementary material, further inquiries can be directed to the corresponding author.

## Ethics statement

The studies involving human participants were reviewed and approved by the Academic Ethics Committee of the School of Psychology at Northeast Normal University. The patients/participants provided their written informed consent to participate in this study.

## Author contributions

BS contributed to the conception and design of the study and wrote sections of the manuscript. ML organized the database, performed the statistical analysis, and wrote the first draft of the manuscript. All authors contributed to the manuscript revision, read, and approved the submitted version.

## Funding

This work was supported by Research on the Improvement of Physical Literacy Level of College Students Reserve Forces in Jilin Province by National Defense Physical Education in the New Era Leader to BS.

## Conflict of interest

The authors declare that the research was conducted in the absence of any commercial or financial relationships that could be construed as a potential conflict of interest.

## Publisher’s note

All claims expressed in this article are solely those of the authors and do not necessarily represent those of their affiliated organizations, or those of the publisher, the editors and the reviewers. Any product that may be evaluated in this article, or claim that may be made by its manufacturer, is not guaranteed or endorsed by the publisher.
